# Views and perceptions of Australian physiotherapists and physiotherapy students about the potential implementation of physiotherapist prescribing in Australia: a survey protocol

**DOI:** 10.1186/s12913-018-3300-x

**Published:** 2018-06-19

**Authors:** T. Noblet, J. Marriot, T. Jones, C. Dean, A. Rushton

**Affiliations:** 1 Centre of Precision Rehabilitation for Spinal Pain (CPR Spine), School of Sport, Exercise and Rehabilitation Sciences, University of Birmingham, Edgbaston, Birmingham, B15 2TT UK; 2 Institute of Clinical Sciences, College of Medical and Dental Sciences, University of Birmingham, Edgbaston, Birmingham, B15 2TT UK; 3 Department of Health Professions, Faculty of Medicine and Health Sciences, Macquarie University, Ground Floor, 75 Talavera Road, Sydney, NSW 2109 Australia

**Keywords:** Non-medical prescribing, Physiotherapy, Australia, Views, Survey, Questionnaire

## Abstract

**Background:**

Non-medical prescribing (NMP) is acknowledged as an expanding area of clinical practice across the world. The physiotherapy profession is currently investigating the introduction of physiotherapist prescribing in Australia, with the case for reform centred around meeting the healthcare needs of the current and future Australian population. Conflict within a profession has been identified as a barrier to implementation of new clinical innovations. An online survey has been developed with the aim to collect and synthesise the views and perceptions of Australian physiotherapists and physiotherapy students about the potential use of NMP by physiotherapists in Australia.

**Methods:**

A cross-sectional descriptive survey design, using a pre-tested online questionnaire, including quantitative and qualitative components, will be utilised to explore the views and perceptions of Australian physiotherapists and physiotherapy students regarding NMP by physiotherapists in Australia. Quantitative data will be analysed descriptively and regression analysis will be utilised to identify associations between the specific question outcomes and demographic data. A thematic analytical approach will be utilised to synthesise qualitative data from open-questions.

**Discussion:**

The results from this survey will serve to inform decision-makers about the current views of the Australian physiotherapy profession with regards to the potential implementation of physiotherapist prescribing in Australia. Data will be used in conjunction with cost-benefit analyses, risk analysis as well as assessment of the health-requirements and consultation with key stakeholders including the Australian health consumer when contemplating change.

**Electronic supplementary material:**

The online version of this article (10.1186/s12913-018-3300-x) contains supplementary material, which is available to authorized users.

## Background

Australian physiotherapists have expressed an interest in non-medical prescribing (NMP) following the introduction of independent physiotherapist prescribing in the United Kingdom (UK) in 2012. Recently, the Australian Physiotherapy Association (APA) in collaboration with the Australia Physiotherapy Council (APC) and Council of Physiotherapy Deans Australia and New Zealand (CPDANZ) have commenced national processes to evaluate potential clinical need, quality and safety issues [1]. The anticipated future implementation in Australia will require physiotherapists, alongside politicians, policy makers and healthcare managers, to welcome change within national and local healthcare systems [1–4]. In July 2015, the APA submitted a proposal for the endorsement of registered physiotherapists for autonomous prescribing to the Physiotherapy Board of Australia [1]. The case for reform centred around meeting the healthcare needs of the modern Australian population. Inequity in access to medicines for people living in rural and remote Australia was recognised as a key driver for the introduction of physiotherapy prescribing. Further, it was suggested that physiotherapy prescribing may also help resolve health equities between Aboriginal and Torres Strait Islander peoples and other minority groups by increased access to medicines via non-medical prescribers (NMPs) in the local communities [1].

A recent mixed methods systematic review of the NMP literature evaluating the facilitators and barriers to NMP, across all professions internationally, identified four main themes affecting the implementation and utilisation of NMP: systems, education and support, personal and professional, and financial factors [5]. Analysis of the ‘Personal and Professional’ theme highlighted that the views and perceptions of individual clinicians’ may or may not agree and/or be synergistic in nature with the overall view of the profession as a whole. This is important as potential conflict within a profession has been identified as a barrier to implementation of new clinical innervations [6]. Anecdotal evidence suggests that physiotherapists prescribing may retain Australian physiotherapists within the profession, as they would be able to optimally utilise their knowledge and skills, providing seamless patient care regardless of geographical location or health sector [1, 7]. However, survey literature investigating the views of NMP professions in the UK and USA suggests that views about NMP by the individual professional may vary depending on the individual’s job specification, access to medical support, geographical location, health sector, level of experience and the timespan of the individual’s career [8–12].

To date no evidence exists evaluating the Australian physiotherapy professions’ views and perceptions about the potential use of NMP by physiotherapists in Australia. If the profession’s views are left unknown, a potential divided opinion within the physiotherapy profession may serve as a barrier to implementation of NMP in the future. For this reason, an online survey has been developed with the aim to collect and synthesise the views of Australian physiotherapists and physiotherapy students about the potential use of NMP by physiotherapists in Australia in order to address the following research question:

What are the views of Australian physiotherapists and physiotherapy students regarding non-medical prescribing (NMP) by physiotherapists in Australia?

More specifically, this study has the following objectives:

1. To explore the views of Australian physiotherapists and physiotherapy students about the potential implementation and use of NMP by physiotherapists in Australia.

2. To explore how the geographical location and health sector that a clinician works/studies in may influence the views of Australian physiotherapists and physiotherapy students about the potential implementation and application of NMP by physiotherapists in Australia.

3. To explore similarities or differences in the views of student physiotherapists and registered physiotherapists of differing years’ experience, about the potential implementation and application of NMP by physiotherapists in Australia.

4. To explore the views of Australian physiotherapists and physiotherapy students about how physiotherapy prescribing might impact the care that the physiotherapy profession can provide.

## Methods/design

To ensure transparency and reproducibility this study protocol follows an adapted version of the Standard Protocol Items: Recommendations for Interventional Trials (SPIRIT) statement [13], in line with the SUrvey Reporting GuidelinE (SURGE) guidance [14]. Unfortunately, no register currently exists for survey research. For this reason, the authors have chosen to publish the study protocol to ensure quality, rigor and transparency [15, 16].

### Survey design

A cross-sectional descriptive survey design, using an online questionnaire will be utilised as this method enables the collection of a broad range of empirical data across a large geographical area in a finite time span [16, 17]. An online questionnaire will be adopted as this can be conducted remotely, enabling participants to complete the survey at a time and place convenient to them, without relying on availability of interviewers, therefore wide spread distribution of the questionnaire to physiotherapists located in all metropolitan, regional and remote areas, across all states and territories of Australia is possible [16, 18].

### Participants

Participant inclusion criteria is outlined in Table 1. Data published by the Physiotherapy Board of Australia reports that 28,855 physiotherapists are currently registered with the Australian Health Professionals Registration Authority (AHPRA) [19]. Currently in Australia there are 20 Universities offering entry level physiotherapy programs with a total of approximately 7000 physiotherapy students enrolled.

**Table 1:** Participant inclusion criteria

• Physiotherapist registered with AHPRA or a student enrolled in an accredited, entry level physiotherapy course in Australia leading to AHPRA registration as a physiotherapist. • Able to read and understand written English. • Able to legally consent to participate in the survey independently.	

### Data collection, management and analysis

#### Procedure

An advertisement containing a link to the online survey will be emailed to all members of the APA on newsletters and associated clinical and professional network’s electronic-communications to encourage participation in the survey. A reminder advert will be sent via email 4 weeks later to facilitate recruitment [16, 18]. Use of the APA membership as a platform for recruitment to this study has been selected as current APA membership is 20,972, representing the majority of physiotherapists and physiotherapy students in Australia [20]. Power calculations have shown a sample of n= > 1037 (95%CI) is required to be representative of the total population [15, 21]. The APA membership is representative of all physiotherapy specialties across all localities in Australia, representing physiotherapists throughout all years of post-qualification practice as well as all student physiotherapists in Australia [20]. It is anticipated that referrals through professional networks will also occur, with participants or professionals who have gained knowledge of the survey communicating the survey’s existence to other registered physiotherapists and/or physiotherapy students [16, 18]. The email link will also be sent to the 20 Universities offering Australian physiotherapy programs via the Council of Physiotherapy Deans Australia and New Zealand for distribution to physiotherapy students. Data collection will take place 1st March - 30th April 2017. Data will be collected automatically by the online survey software, Qualtrics (Qualtrics, Provo, UT) to avoid human data inputting errors [22].

#### The questionnaire

The questionnaire was designed using evidence from a thematic synthesis of data from a mixed methods systematic review examining the barriers to and facilitator of NMP [5]. The review identified that the personal views of members of a profession utilising NMP were key to the implementation of NMP by that profession. To ensure the inclusion of the optimal questions the views, knowledge and perceptions of non-medical prescribers (NMPs) from a variety of professions internationally highlighted in the systematic review were prioritised through consultation with experts in the fields of physiotherapy, non-medical prescribing and Australian state/federal law and health policy [16, 17, 23]. The included questions were designed to specifically answer the research objectives [16, 17, 23].

The online survey was built using Qualtrics Research Suite survey software (Qualtrics, Provo, UT). This software was selected as it enables online questionnaires to be completed on a range of electronic devices, including both desktop, laptop and mobile based devices, whilst storing data in real-time [24]. Context specific questioning is utilised to limit acquiescence bias [25]. To minimise the difficulty of the survey for participants and combat potential satisficing, we have aimed to minimise duration and distractions via fluidity of design and inbuilt survey logic [26]. A short survey (5–10 min completion time) has been designed containing one question per page to maximise recruitment [22].

#### Questions/measures

The full questionnaire can be found in Additional file 1. In summary, the questionnaire comprises of four sections of questions consisting specifically of:

Section 1: Demographics.

This section contains 11 closed demographic questions regarding the participants age, gender, level of experience, clinical specialty and locality (reported using the Rural, Remote and Metropolitan Areas (RRMA) classification [27].

Section 2: Views about the physiotherapy profession.

The second section contains four closed-answer questions regarding the participants views about NMP by physiotherapists, giving opportunity to express opinions regarding benefits and/or concerns [5].

Section 3: Views about the individual physiotherapist.

The third section contains three closed-answer questions designed to collect data regarding the likelihood of the individual participants to train as a prescriber and their motivations/ barriers to do this should physiotherapist prescribing become a legal reality in Australia [5]. Inbuilt survey logic ensures participants are shown only those questions that are pertinent to them based on their previous answers provided.

Section 4: Wider impacts.

Section 4 contains two open-ended questions designed to gather qualitative data regarding the participants’ views and perceptions about how physiotherapist prescribing might positively or negatively impact on the care that the profession can provide to all patient groups. Open questions are utilised to allow the participants to express individualised answers to complex questions without limitation [16]. The final question also allows participants to share any additional information that they deem applicable and relevant to the survey, aiming to capturing useful insight not considered elsewhere within the questionnaire [16, 17].

#### Pre-testing

The questionnaire was piloted by a sample of the target population (*n* = 10) to test for internal consistency [17]. The pilot participants are not excluded from completing the full questionnaire. Ten participants were purposely sampled representing the physiotherapy professional population in Australia, including key specialties and student physiotherapists [16, 17]. The pre-testing followed the procedure for the main survey to enable the identification of potential problems with interpretation of the instructions and questions, and identification of any potential reasons for poor responses [16, 17]. Following the pre-testing, small changes were made to the survey logic to optimise the user experience, and Anglo-Australian terminology was clarified to minimise confusion due to linguistics.

#### Data storage

All data will be electronic and stored in password protected computer files that can be accessed only by study investigators at Macquarie University and the University of Birmingham. Participants who choose to disclose personal details will be additionally protected via coding on data files. This coding will be kept in a password protected computer file on the University of Birmingham and Macquarie University servers, only accessible to the research team ensuring confidentiality [16, 17]. The password-protected files will be retained for 10 years, satisfying policies at both the University of Birmingham and Macquarie University.

#### Data analysis

Only data from completed questions from fully completed questionnaires will be included in the data analysis. Demographic data will be tabulated and primary descriptive analysis of the data will be completed [16]. For the data collected from questions in sections 2 and 3, we will utilise multinomial regression for questions with a single option response and poisson regression for multiple-option questions, to determine the likelihood that health sector, geographical location, or years qualified as a physiotherapist, are associated with specific views. To ensure the transparent synthesis of data from the questions in section 4, we will use thematic analysis to identify key themes in the data [28]. Answers will be coded line-by-line using NVivo 11 software (QSR International, Melbourne, Australia) by one researcher (TN) and be verified by a second researcher (TJ). Independently generated themes/sub-themes will then be discussed with a panel of experts for confirmation and agreement [28].

### Ethics and dissemination

#### Ethical considerations

To ensure that the survey is conducted in an ethical manner within best research practice, ethical approval was sort [16, 17]. Approval was granted on 5th December 2016, by the Medical Sciences Human Research Ethics Committee (HREC), Macquarie University, Australia (Reference No**:** 5201600846), and verified by the Research Governance Officer at the University of Birmingham, UK, on the 12th December 2016 (Reference No: ERN_16–1576).

#### Consent to participate

Completion of the survey via the link will be entirely voluntary, with no incentives offered to participants to minimise bias [16, 23]. Participant consent will be gained using an online consent form following the provision of information explaining the rationale, content and research dissemination plans to ensure ethical recruitment of participants (the online information and consent for can be found in Additional file 2) [16, 17]. This information and consent section is situated at the start of the online questionnaire. A response to the online consent question will be required before participants can progress to the study questions. Any participants who select the ‘no consent’ option will automatically exit the questionnaire. Contact details for the research team will be provided to give the participants the opportunity to have any questions they have answered [16, 23]. Participants will be able to stop completing the survey at any point [16, 17]. All surveys will be anonymous unless personal information is disclosed by the participants [17].

#### Dissemination of findings

The study’s findings will be disseminated via study reports, publication in academic peer-reviewed journals and conference presentations [16]. The results will be communicated to participants on request as a summary report written in lay language including key findings and plans for future research.

## Discussion

The results from this survey will serve to inform decision-makers about the current views of the Australian physiotherapy profession with regards to the potential implementation of physiotherapist prescribing in Australia. Evidence is required by the physiotherapy professional association, health departments and political leaders to inform clinically safe and economically sound decisions about redefining the scope of physiotherapy in Australia to include NMP. Innovation in professional scope requiring the amendment of legislation and regulation is costly and time consuming [1–4]. Change is seen to be facilitated by a benefit to the health economy and improvements in health care to service users [1, 4]. Resistance to change within the profession is reported as a barrier to implementation of new clinical innovations [5]. The results of this study should be used in conjunction with cost-benefit analyses, risk analysis as well as assessment of the health-requirements and consultation with key stakeholders including the Australian health consumer when contemplating change. It is anticipated that observations identified from the data may inform further research. Future focus groups and interviews with physiotherapist and other stakeholders may be utilised to investigate specific observations of interest in detail, enhancing data richness [16, 29].

## Additional files

Additional file 1: Online Questionnaire. (DOCX 19 kb)

Additional file 2: Online Information and Consent Form. (DOCX 16 kb)

## Abbreviations

AHPRA: Australian Health Professionals Registration Authority
APA: Australian Physiotherapy Association
APC: Australia Physiotherapy Council
CPDANZ: Council of Physiotherapy Deans Australia and New Zealand
HREC: Human Research Ethics Committee
NMP: Non-medical prescribing
NMPs: Non-medical prescribers
RRMA: Rural, Remote and Metropolitan Areas classification
UK: United Kingdom
