# Supplementary material for: Views and perceptions of Australian physiotherapists and physiotherapy students about the potential implementation of physiotherapist prescribing in Australia: a survey protocol
Source: BMC Health Serv Res. 2018 Jun 19;18:472. doi: 10.1186/s12913-018-3300-x (PMC6006587; doi:10.1186/s12913-018-3300-x)
Supplement: Supplementary file 1 — Online Questionnaire. (DOCX 19 kb) [file 12913_2018_3300_MOESM1_ESM.docx]

Additional file 1: Online Questionnaire

Q1 What is your gender?

- Male (1)
- Female (2)
- Other (3)

Q2 What is your age?

- 17-29 (1)
- 30-39 (2)
- 40-49 (3)
- 50-59 (4)
- 60 or older (5)

Q3 Which of the following are you?

- AHPRA registered physiotherapist (1)
- Student physiotherapist enrolled in an Australian university (2)

If Student physiotherapist enr... Is Selected, Then Skip To Which state or territory do you curre...

Q4 How many years have you been a qualified physiotherapist?

- 0-4 (1)
- 5-9 (2)
- 10-14 (3)
- 15-19 (4)
- 20 or more (5)

Q5 Where did you obtain your primary physiotherapy qualification?

- Australia (1)
- Overseas (please specify) (2) ____________________

Q6 Which state or territory do you currently work? If multiple, select the state or territory that you spent the most time working in over the past 14 days.

- Australian Capital Territory (1)
- New South Wales (2)
- Northern Territory (3)
- Queensland (4)
- South Australia (5)
- Tasmania (6)
- Victoria (7)
- Western Australia (8)

Q7 Do you work in a metropolitan or rural area?    Please choose the most appropriate option. If you work in multiple areas, select the area in which you spent the most hours working in the past 14 days.   If you are unsure, you can check your areas classification using the following website: http://www.doctorconnect.gov.au/internet/otd/publishing.nsf/Content/locator

- RA1 - Major Cities of Australia (1)
- RA2 - Inner Regional Australia (2)
- RA3 - Outer Regional Australia (3)
- RA4 - Remote Australia (4)
- RA5 - Very Remote (5)

Q8 In which health sector do you spend most of your time working as a physiotherapist?

- Public sector (1)
- Private sector (2)
- Educational/research institute or university (3)
- Not-for-profit organisation (4)
- Other (please specify) (5) ____________________

Q9 What area/s of physiotherapy do you predominantly work in or identify with?Please select up to a maximum of three (3) areas.

- Amputees (1)
- Burns/plastics (2)
- Cardiorespiratory/acute medicine/surgery (3)
- Chronic disease management (4)
- Education (5)
- Emergency department (6)
- Gerontology/Aged care (7)
- Health promotion/Public health (8)
- Lymphoedema (9)
- Mental health (10)
- Musculoskeletal/orthopaedics (11)
- Neurology (12)
- Occupational health (13)
- Paediatrics (14)
- Pain (15)
- Palliative care (16)
- Rehabilitation (mixed) (22)
- Rheumatology (17)
- Rural generalist (18)
- Sports (21)
- Women's health/continence (19)
- Veterinary (20)

Display This Question:

If Which of the following are you? Student physiotherapist enrolled in an Australian university Is Selected

Q10 Which state or territory do you currently attend university?

- Australian Capital Territory (1)
- New South Wales (2)
- Northern Territory (3)
- Queensland (4)
- South Australia (5)
- Tasmania (6)
- Victoria (7)
- Western Australia (8)

Q11 Autonomous prescribing: "Prescribing occurs where a prescriber undertakes prescribing within their scope of practice without the approval or supervision of another health professional. The prescriber has been educated and authorised to autonomously prescribe in a specific area of clinical practice. Although the prescriber may prescribe autonomously, they recognise the role of all members of the health care team and ensure appropriate communication occurs between team members and the person taking medicine".  The Health Professionals Prescribing Pathway (HPPP), p16 (2013)   To what extent do you agree with the following statement:   "I believe that autonomous prescribing responsibilities should be introduced for physiotherapists in Australia."

- Strongly agree (1)
- Agree (2)
- Neither agree nor disagree (3)
- Disagree (4)
- Strongly disagree (5)

Q12 What do you see the benefits of physiotherapists prescribing medicines to be?Select as many options as are appropriate to you.

- Improved efficiency of service delivery (1)
- Reduced costs of health care delivery to the consumer (2)
- Improved consumer experience (3)
- Reduction in the overall costs of healthcare to the Australian economy (4)
- Improved retention of clinicians within the physiotherapy profession (5)
- Potential for enhanced remuneration (6)
- Reduced safety risks to consumers (7)
- Improved access for consumers to prescription medications (8)
- Future proofing the Australian healthcare system with a flexible workforce (9)
- Other (please specify) (10) ____________________
- I do not believe there would be any benefits (11)

Q13 What are your concerns about the prescription of medicines by physiotherapists?Select as many options as are appropriate to you.

- Prescribing of medicines is not a physiotherapists' role (1)
- Physiotherapists do not have adequate pre-requisite knowledge to undertake a prescribing course (2)
- There is no need for physiotherapists to prescribe medicines (3)
- Physiotherapist prescribing will create a two (2) tier profession (4)
- Physiotherapist prescribing will increase safety risks to consumers (5)
- Remuneration does not match the responsibility associated with the prescribing of medicines (6)
- Other (please specify) (7) ____________________
- I do not have any concerns (8)

Q14 How many years experience do you think a physiotherapist should have prior to being able to train as a physiotherapist prescriber?

- 0 - Should be included in pre-registration physiotherapy qualification (1)
- 1-2 years (2)
- 3-5 years (3)
- 6-9 years (4)
- 10 or more years (5)
- Physiotherapists should not be able to train as prescribers (6)

Q15 If physiotherapists became able to autonomously prescribe medicines, how likely are you to want to train to become a prescriber?

- Extremely likely (1)
- Somewhat likely (2)
- Neither likely nor unlikely (3)
- Somewhat unlikely (4)
- Extremely unlikely (5)

Display This Question:

If If physiotherapists became able to autonomously prescribe medicines, how likely are you to want t... Extremely likely Is Selected

Or If physiotherapists became able to autonomously prescribe medicines, how likely are you to want t... Somewhat likely Is Selected

Or If physiotherapists became able to autonomously prescribe medicines, how likely are you to want t... Neither likely nor unlikely Is Selected

Q16 What are your key motivations to becoming a prescriber?Select as many options as are appropriate to you.

- Improving the care I am able to provide (1)
- Improved job satisfaction (2)
- Increased remuneration (3)
- Improved professional reputation (4)
- Other (please specify) (5) ____________________

Display This Question:

If If physiotherapists became able to autonomously prescribe medicines, how likely are you to want t... Extremely unlikely Is Selected

Or If physiotherapists became able to autonomously prescribe medicines, how likely are you to want t... Somewhat unlikely Is Selected

Or If physiotherapists became able to autonomously prescribe medicines, how likely are you to want t... Neither likely nor unlikely Is Selected

Q17 What makes you unlikely to want to train as a prescriber?Select as many options as are appropriate to you.

- I do not believe that physiotherapists should prescribe medicines (1)
- I do not think that I have the knowledge required to train as a prescriber (2)
- I do not wish to complete additional training (3)
- I am not prepared to take on the additional responsibility associated with prescribing medicines (4)
- In my current role, being able to prescribe would not change the care provided (5)
- A prescriber is readily available to the clients that I provide care for (6)
- I work in a non-clinical role (7)
- Other (please specify) (8) ____________________

Q18 Do you have any additional thoughts about how physiotherapist prescribing may impact the care that the profession is able to provide? For example a positive or negative impact on a specific group e.g. minority groups, immigrants, students, travellers......

Q19 Is there any additional information you would like to share at this time?
