# Supplementary material for: Views and perceptions of Australian physiotherapists and physiotherapy students about the potential implementation of physiotherapist prescribing in Australia: a survey protocol
Source: BMC Health Serv Res. 2018 Jun 19;18:472. doi: 10.1186/s12913-018-3300-x (PMC6006587; doi:10.1186/s12913-018-3300-x)
Supplement: Supplementary file 2 — Online Information and Consent Form. (DOCX 16 kb) [file 12913_2018_3300_MOESM2_ESM.docx]

Additional file 2: Online Information and Consent Form

Non-medical prescribing by physiotherapists in Australia

Thank you for taking the time to visit this site. This page provides detailed information about this survey. Please take the time to read this information carefully.

What does the survey aim to do?

We are interested in the thoughts and beliefs of Australian physiotherapists and physiotherapy students regarding about the potential use of non-medical prescribing by physiotherapists in Australia.

(NB, non-medical prescribing is the prescribing of medicines by professionals other than medical doctors)

What is involved?

If you decide to participate, you will be asked to complete a brief survey that will involve answering questions regarding your thoughts and beliefs regarding non-medical prescribing by physiotherapists in Australia. We estimate that this survey will take approximately 5-10 minutes.

Who can participate in this survey?

Anyone who is 17 years and over, and who is a registered physiotherapist with the Australian Health Practitioner Regulation Agency (AHPRA), or is a student studying physiotherapy at an Australian university is eligible to participate in this survey.

Are there any risks?

There are no anticipated risks associated with undertaking this survey. Participation in this study is entirely voluntary. You are not obliged to participate in or to complete this survey.

What about privacy and confidentiality?

Participants will remain anonymous at all times during this survey. Basic demographic details will be collected but this will not be of a nature as to allow for identification. All data will be stored in password protected computer files that can be accessed only by study investigators at Macquarie University. Any data generated from this survey used in future research studies will only be done so in non-identifiable form.

Who is conducting this survey?

This study is being carried out by physiotherapist, Tim Noblet, as part of a larger body of research being conducted to meet the requirements of Doctor of Philosophy in Physiotherapy. Tim is working with Dr Taryn Jones and Professor Catherine Dean from Macquarie University, as well as Dr Alison Rushton and Professor John Marriott from the University of Birmingham, UK.

The ethical aspects of this study have been approved by the Macquarie University Human Research Ethics Committee. If you have any complaints or reservations about any ethical aspect of your participation in this research, you may contact the Committee through the Director, Research Ethics (Ph: +61 2 9850 7854; email: ethics@mq.edu.au).

Can I contact the researchers?

The researchers can be contacted by email on tim.noblet@mq.edu.au or by phone on +61 02 9850 6614.

Am I able to obtain a summary of the study results?

Yes, a short summary of the overall study results will be available once the study has been completed. Should you be interested in obtaining a copy of this summary please email Tim Noblet on tim.noblet@mq.edu.au.

What do I do now?

If you have read and understood the above information and would like to participate in the survey please select "yes" below knowing you can withdraw from the survey at any stage. Should you not wish to participate further, you can select "no" and we thank you for your time.

- Yes, I have read and understood the information provided to me and would like to participate in the survey. (1)
- No, I would prefer to not participate in this survey. (2)

If No, I would prefer to not p... Is Selected, Then Skip To End of Survey
